# Porcine Corneas Incubated at Low Humidity Present Characteristic Features Found in Dry Eye Disease

**DOI:** 10.3390/ijms23094567

**Published:** 2022-04-20

**Authors:** Alice Rocha Teixeira Netto, José Hurst, Karl-Ulrich Bartz-Schmidt, Sven Schnichels

**Affiliations:** Centre for Ophthalmology, Clinical Research University Eye Hospital Tübingen, Eberhard Karls Universität Tübingen, Elfriede-Aulhorn-Straße 7, D-72076 Tübingen, Germany; alicerochanetto@hotmail.com (A.R.T.N.); u.bartz-schmidt@uni-tuebingen.de (K.-U.B.-S.); sven.schnichels@med.uni-tuebingen.de (S.S.)

**Keywords:** dry eye disease, porcine model, corneal epithelium, ex vivo model, dry eye disease treatment

## Abstract

Dry eye is a multifactorial disease that affects the ocular surface and tear fluid. Current treatment options include lubricant eye drop application several times a day. However, these eye drops often cause local side effects like ocular allergies or blurred vision after the application. To test new treatment options, a robust dry eye model is needed. Here, a porcine ex vivo model was established by means of incubation of porcine corneas in low humidity (LH) and characterized by histological damage evaluation, epithelial thickness and by relevant dry eye markers, such as interleukin 1 beta (IL-1β), nuclear factor kappa-light-chain-enhancer of activated B cells (NF-κB), occludin and galectin-3. In the dry eye model proposed, an increased secretion of IL-1β was observed, as well as an upregulation of *NF-κB*, *occludin* and *galectin-3* mRNA expression. Moreover, the model presented a higher rate of cell death in comparison to the controls. These effects could be reversed with successful treatment of dexamethasone (dexa) and partially reversed with hyaluronic acid (HA) containing eye drops. Furthermore, medium-molecular-weight HA stimulated an increase in IL-1β in the model proposed. In conclusion, this dry eye model mimics the in vivo condition and hence allows for animal-free testing of novel dry eye treatments.

## 1. Introduction

Dry eye is a multifactorial disease that affects the ocular surface and tear fluid [1]. Typical symptoms include foreign body sensation in the eye, burning eyes, itching and photosensitivity [2]. The prevalence of dry eye in the general population is 5–35% worldwide, depending on the age group [1]. Current treatment options for this chronic eye disease mainly include application of eye drops several times a day [3]. However, these eye drops often cause local side effects like ocular allergies or blurred vision after the application, which leads to a reduced compliance [4]. To test new treatment options, a robust dry eye model is needed.

The cornea is a transparent tissue without blood vessels and forms a structural barrier [5]. The most external layer of the cornea is the corneal epithelium, in which cells are connected by tight junctions. The dense apical glycocalyx at the corneal epithelium is rich in transmembrane mucins and plays an important role as barrier function, lubricant and helps to combat microbial colonization [6]. The epithelial glycocalyx is composed of transmembrane mucins (MUC), such as MUC1, MUC4 and MUC16 and a soluble lectin, galectin-3. Structurally, galectin-3 has carbohydrate recognition domains (CRD) that bind to MUC1 and MUC16 and form a galectin–glycoprotein lattice. This complex is responsible for a protective barrier function and also for the modulation of cell–cell, cell–matrix and cell–pathogen interaction [6,7].

Tight junctions are formed from two protein classes. The transmembrane proteins, which connect the membranes of the neighbor cells and the tight junction-associated proteins. The tight junction-associated proteins are intracellular proteins and lie on the cytoplasmic side. The transmembrane proteins include occludin, claudin and junctional adhesion molecule (JAM). The proteins also known as the Zonula occludens proteins (ZO-1, ZO-2, ZO-3) are responsible for binding the transmembrane protein and the cytoskeleton, ensuring that the tight junction is well fixated [8].

Studies indicate that chronic dry eyes are characterized by increased osmolarity of the tear film and inflammatory processes that affect the lacrimal gland and the corneal and conjunctival epithelia [9]. Briefly, the hyperosmolarity of the tear film or the drying out of the ocular surface leads to the production of pro-inflammatory cytokines, matrix metalloproteinases and pro-apoptotic factors, ultimately leading to cell death [10,11,12]. In addition, activated matrix metalloproteinases decompose the extracellular matrix and thus cause a loss of cell–cell adhesion [11]. Together, these processes lead to the loss of stability and integrity of the cornea.

Desiccating stress and hyperosmolarity of tear fluid stimulate an innate immune pathway by activation of NLR family pyrin domain containing 3 (NLRP3) inflammasome. NLRP3 ultimately leads to maturation and secretion of the pro-inflammatory cytokine interleukin 1 beta (IL-1β) [13]. Nuclear factor kappa-light-chain-enhancer of activated B cells (NF-κB) is involved in the cellular response to stimuli such as stress, cytokines, and free radicals and stimulates secretion of IL-1β and tumor necrosis factor alpha (TNF-α). Both IL-1β and TNF-α activate matrix metalloproteinase 9 (MMP9) [12]. MMP9 cleaves tight junctions’ components such as occludin and ZO-1 and galectin-3 [7]. These factors cause loss of epithelial corneal cells and increase permeability of corneal epithelium.

In vivo dry eye models have been already proposed, for example, based on the surgical removal of the lacrimal gland or on mechanical inhibition of blinking, causing dehydration to the eye surface [14]. Tear secretion inhibition made by drugs has also been proposed [14,15]. However, the use of animals for research purposes should be reduced due to, ethical issues, which makes the development of a simple and easily reproducible ex vivo model for dry eye disease (DED) needed. 

Although several ex vivo models have been proposed previously, they are either laborious or difficult to set up, thus reducing the reproducibility and efficiency. For instance, whole porcine eyes can be obtained from a local abattoir and fixed in a holder. Simulating the flow of tears and blinking at long intervals using a ventilator can reproduce dry eyes. To simulate blinking and lacrimation, a computer-controlled mechanical arm system has also been explored in the literature. It was able to move the eyelid of the porcine eye and apply phosphate buffered saline (PBS). On the other hand, this apparatus makes the study more variable due to increased number of factors involved, and more expensive [16].

In this study, an easy-to-reproduce ex vivo model that offers the unique possibility of viewing the entire tissue complex of a cornea while being able to better mimic the in vivo conditions, thus avoiding in vivo experiments, is established and characterized by suitable markers. It is important to note that ex vivo experiments still use animal tissue, but as the eyes are received from local abattoirs, the animals are primarily slaughtered for nutritional purposes.

To treat DED, the use of artificial tears composed of hyaluronic acid (HA) and the use of corticosteroids, such as dexamethasone (dexa), is widespread. It has already been shown that corticosteroids present a therapeutic effect against the inflammation processes that occur at DED [17,18]. Moreover, the efficacy of HA to moisture the eye surface and to improve DED symptoms was already demonstrated [15,19]. Therefore, dexamethasone 1.3 mg/mL (DexaEDO^®^, Bausch-Lomb, Heidelberg, Germany) and hyaluronic acid 0.2% (Artelac^®^ Splash EDO^®^, Bausch-Lomb, Heidelberg, Germany) were used in this study to investigate whether it would be therapeutically effective in the porcine dry eye model proposed.

## 2. Results

After corneal extraction, porcine corneas were incubated at LH for 12 h, 24 h or 48 h, and their controls were incubated at high humidity (HH) for the same period, respectively.

### 2.1. Low Humidity Causes Corneal Damage and Corneal Thinning

**Figure 1:**
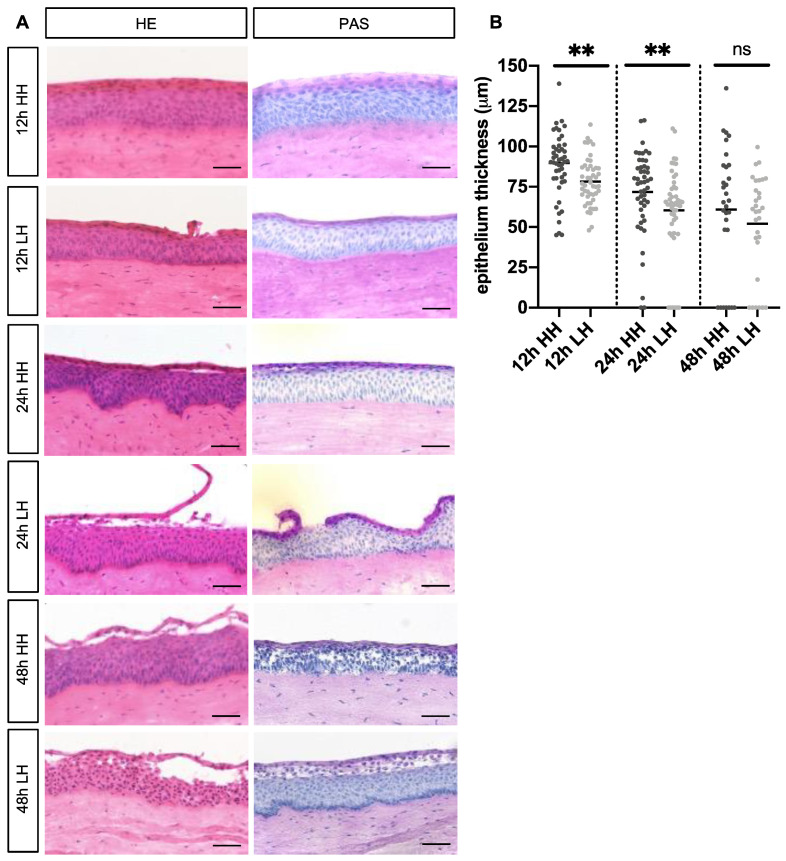
Low humidity (LH) causes corneal damage and corneal thinning. (**A**) Representative images of porcine corneas subjected to LH and high humidity (HH), respectively, for different time points. Samples incubated at LH for 12 h stained with HE and PAS exhibited small desquamation of surface corneal epithelial cells in comparison to controls. Samples submitted to LH for 24 h stained with HE and PAS demonstrated epithelial damage and detachment of glycoprotein layer when compared to HH samples. Samples submitted to LH and to HH for 48 h stained with HE and PAS demonstrated epithelial damage and detachment of glycoprotein layer. (**B**) Epithelial thickness of samples incubated at LH for 12 h (78.29 µm ± 2.11) was significantly reduced in comparison to the 12 h HH group (89.77 µm ± 3.17; *p* = 0.003) (*n* = 8 for 12 h LH/*n* = 8 for 12 h HH). Moreover, epithelial thickness of samples submitted to LH for 24 h (60.34 µm ± 3.98) was significantly reduced when compared to their controls (71.80 µm ± 3.75; *p* = 0.009) (*n* = 7 for 24 h LH/*n* = 8 for 24 h HH). No significant different was observed between samples submitted to LH (52.09 µm ± 5.71) and HH (60.90 µm ± 7.21; *p* = 0.342) for 48 h (*n* = 5 for 48 h LH/*n* = 5 for 48 h HH). Scale bar = 50 µm. LH: low humidity. HH: high humidity. HE: Hematoxylin-Eosin. PAS: Periodic acid–Schiff. ns: not significant. All data are shown as mean ± SEM. ** *p* < 0.01.

To evaluate histological changes caused by LH (30% humidity), HE and PAS staining were performed, while controls were incubated at HH (95% humidity). Histological examination revealed that the LH treated group for 12 h exhibited small desquamation of surface epithelial cells in comparison to the control group. However, detachment of the superficial cells was observed in porcine corneas incubated at LH for 24 h compared to controls (Figure 1A). The glycoprotein layer covering the apical epithelium was detaching from the apical layer when subjected to LH for 24 h, but this detachment could not be observed in the control group which was incubated at HH (Figure 1A). Moreover, the epithelium thickness was reduced in samples treated with LH for 12 h (78.29 µm ± 2.11) and for 24 h (60.34 µm ± 3.98) in comparison to the ones submitted to HH for 12 h (89.77 µm ± 3.17; *p* = 0.003) and for 24 h (71.80 µm ± 3.75; *p* = 0.009) (Figure 1B). 

To investigate whether a longer period of incubation at LH would also induce changes found in DED, such as detachment of corneal epithelial cells and corneal thinning, porcine corneas were incubated at LH for 48 h. Not only detachment of the superficial corneal cells, but also damage of deeper epithelium layers was observed. Similar changes were also found in controls (Figure 1A). Furthermore, no significant difference was found in epithelial thickness in samples incubated at LH (52.09 µm ± 5.71) for 48 h and HH (60.90 µm ± 7.21; *p* = 0.342) (Figure 1B), probably because both stressed samples and their controls presented severe epithelial damage.

### 2.2. Low Humidity Induces Inflammation in Porcine Model

**Figure 2:**
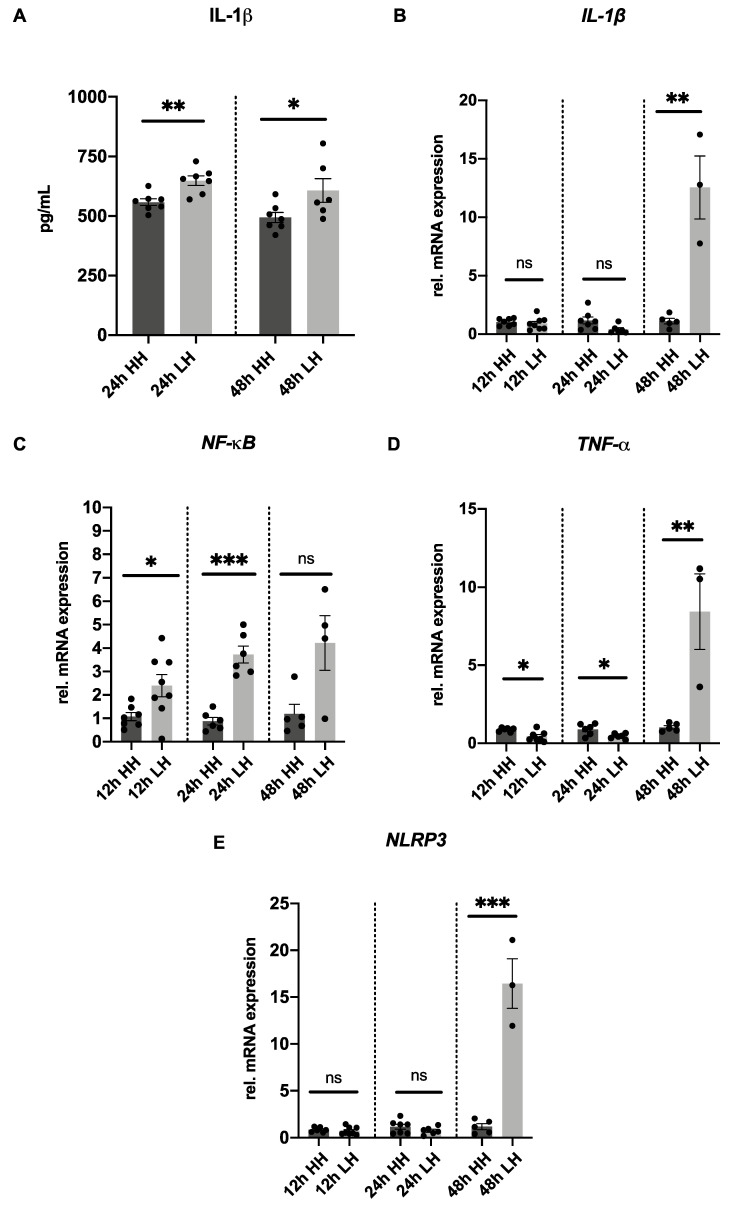
LH induces expression of inflammatory markers after LH treatment compared to incubation with HH. (**A**) A significantly higher secretion of IL-1β was observed in porcine corneas submitted to LH for 24 h (648.30 pg/mL ± 20.30) when compared to 24 h HH (558.20 pg/mL ± 14.50; *p* = 0.004) (*n* = 7 for 24 h LH/*n* = 7 for 24 h HH) and for 48 h LH (606.90pg/mL ± 49.35) in comparison to 48 h HH (494.00 pg/mL ± 21.31; *p* = 0.05) (*n* = 6 for 48 h LH/*n* = 7 for 48 h HH). (**B**) No change of *IL-1β* mRNA expression was observed in corneas incubated at LH for 12 h (0.90 ± 0.19; *p* = 0.58) and for 24 h (0.40 ± 0.15; *p* = 0.05) in comparison to their controls. *IL-1β* expression was 12.54-fold upregulated in samples incubated at LH for 48 h (12.54-fold ± 2.69; *p* = 0.001). (**C**) Gene expression of *NF-κB* was 2.40-fold (±0.48; *p* = 0.03) upregulated in samples cultivated at LH for 12 h and 3.73-fold (±0.36; *p* < 0.001) upregulated in the ones incubated at LH for 24 h. A non-significant increase was observed between samples incubated at LH for 48 h (4.22 ± 1.17; *p* = 0.07) compared to corresponding HH samples. (**D**) *TNF-α* mRNA was downregulated in samples incubated at LH for 12 h (0.44 ± 0.12; *p* = 0.01) and for 24 h (0.45 ± 0.07; *p* = 0.03) in comparison to the corresponding HH samples. *TNF-α* mRNA was 8.44-fold upregulated in samples cultivated at LH for 48 h (±2.42; *p* = 0.006). (**E**) Corneas exposed to LH for 12 h (0.72 ± 0.14; *p* = 0.54) and for 24 h (0.73 ± 0.16; *p* = 0.22) presented no significant difference in *NLRP3* expression in comparison to their HH controls. *NLRP3* expression was 16.43-fold (±2.65; *p* < 0.001) upregulated in samples incubated at LH for 48 h. *n* = 7–8 for 12 h LH/*n* = 6–7 for 12 h HH/*n* = 6 for 24 h LH/*n* = 6–7 for 24 h HH/*n* = 3–4 for 48 h LH/*n* = 4–6 for 48 h HH. LH: low humidity. HH: high humidity. ns: not significant. The bars represent the mean values, and the SEM is shown. * *p* ˂ 0.05 ** *p* ˂ 0.01 *** *p* ˂ 0.001.

Since DED is accompanied with inflammatory process, IL-1β secretion was measured to detect inflammatory process triggered by LH. An increased secretion of IL-1β was evident in the LH group incubated for 24 h (648.30 pg/mL ± 20.30) compared to HH (558.20 pg/mL ± 14.50; *p* = 0.004). This increase in the inflammatory marker IL-1β was also detected in samples exposed to LH stress for 48 h (606.90 pg/mL ± 49.35) when compared to HH samples (494.00 pg/mL ± 21.31; *p* = 0.05) (Figure 2A).

To confirm that inflammation is induced in the model proposed, further inflammatory markers were analyzed. mRNA expression of the pro-inflammatory markers *NF-κB, TNF-α*, *IL-1β* and the inflammasome marker *NLRP3* were evaluated. *IL-1β* expression was 12.54-fold (±2.69; *p* = 0.001) upregulated in samples incubated at LH for 48 h. However, no significant difference was observed in *IL-1β* expression in corneas incubated at LH for 12 h (0.90 ± 0.19; *p* = 0.58) and for 24 h (0.40 ± 0.15; *p* = 0.05) in comparison to their controls (Figure 2B). The mRNA expression of *NF-κB* was 2.40-fold (±0.48; *p* = 0.03) upregulated in samples cultivated at LH for 12 h and 3.73-fold (±0.36; *p* < 0.001) upregulated in the ones incubated at LH for 24 h compared to the corresponding HH corneas. A non-significant increase was found in *NF-κB* expression in corneas submitted to 48 h at LH (4.22 ± 1.17; *p* = 0.07) in comparison with 48 h at HH samples (Figure 2C). Regarding mRNA expression of *TNF-α,* it was 8.44-fold (±2.42; *p* = 0.006) upregulated in porcine corneas exposed to LH for 48 h. A significant downregulation in *TNF-α* expression was shown in samples exposed to 12 h at LH (0.44 ± 0.12; *p* = 0.01) and to 24 h at LH (0.45 ± 0.07; *p* = 0.03) in comparison to their controls (Figure 2D). After 12 h and 24 h, there was no significant difference in corneas exposed to LH in the *NLRP3* expression observed (12 h LH= 0.72 ± 0.14; *p* = 0.54/24 h LH = 0.73 ± 0.16; *p* = 0.22), but after 48 h, *NLRP3* was 16.43-fold (±2.65; *p* < 0.001) upregulated in samples incubated at LH in comparison to their respective controls (Figure 2E).

Furthermore, the protein levels of pro-inflammatory cytokines were investigated in the culture media of stressed samples and detected with a cytokine array. An increase in pro-inflammatory cytokines in the culture media of samples incubated at LH was verified. Interferon-gamma (IFN-γ) and macrophage migration inhibitory factor (MIF) were increased in the media of samples incubated at LH for 48 h (2.67-fold for INF-γ and 1.68-fold for MIF). Fibroblast growth factor 21 (FGF21), which stimulates angiogenesis, and INF-β, which can have either a pro- or anti-inflammatory effect, were increased in the media of porcine corneas incubated at LH for 24 h (4.92-fold for INF-β and 1.54-fold for FGF21). In addition, angiopoietin-1 (ang1), which induces angiogenesis and inflammation, was increased in the media of corneas stressed at LH for 48 h (2.10-fold for ang1) (Table 1).

Therefore, it can be assumed that inflammation was activated in the porcine corneas submitted to LH stress for 24 h and for 48 h compared to the matching HH samples.

### 2.3. Low Humidity Upregulates Tight Junction and Glycocalyx Markers

**Figure 3:**
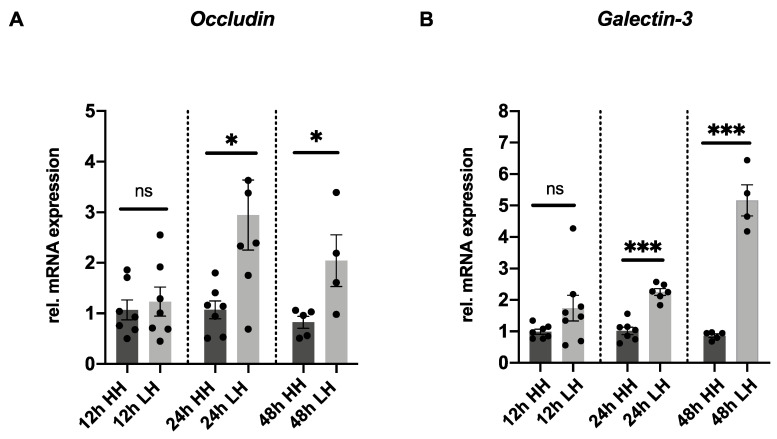
LH stimulates mRNA upregulation of tight junction and glycocalyx markers. (**A**) *Occludin* mRNA expression was 2.94-fold (±0.69; *p* = 0.02) upregulated in porcine corneas incubated at LH for 24 h and 2.56-fold (±0.64; *p* = 0.04) upregulated at the 48 h LH group. No difference in *occludin* expression was identified in porcine corneas incubated at LH for 12 h (1.23 ± 0.29; *p* = 0.65) in comparison to the 12 h HH controls. (**B**) *Galectin-3* mRNA expression was 2.25-fold (±0.11; *p* < 0.001) upregulated in corneas incubated at LH for 24 h and 5.16-fold (±0.49; *p* < 0.001) upregulated in the ones cultivated at LH for 48 h. No difference in *galectin-3* expression was verified in porcine corneas incubated at LH for 12 h (1.74 ± 0.41; *p* = 0.12) in comparison to the 12 h HH controls. *n* = 8 for 12 h LH/*n* = 7 for 12 h HH/*n* = 6–7 for 24 h LH/*n* = 7 for 24 h HH/*n* = 4 for 48 h LH/*n* = 5 for 48 h HH. LH: low humidity. HH: high humidity. The bars represent the mean values, and the SEM is shown. * *p* ˂ 0.05 *** *p* ˂ 0.001. ns: not significant.

*Occludin* mRNA expression has been described as an early marker that predicts the severity of corneal damage [33]. Therefore, to further investigate why epithelial cells detach from corneal epithelial, especially in the samples submitted to LH, mRNA expression of the tight junctions’ marker, *occludin*, was analyzed. Moreover, mRNA expression of the glycocalyx marker, *galectin-3*, was performed. A 2.94-fold (±0.69; *p* = 0.02) upregulation of *occludin* mRNA expression was observed in samples subjected to LH stress for 24 h and a 2.56-fold (±0.64; *p* = 0.04) upregulation was shown in the 48 h LH group compared to the corresponding HH samples. No difference in *occludin* expression was identified in porcine corneas incubated at LH for 12 h (1.23 ± 0.29; *p* = 0.65) in comparison to the 12 h HH controls (Figure 3A). *Galectin-3* mRNA was 2.25-fold (±0.11; *p* < 0.001) upregulated in samples incubated at LH for 24 h and 5.16-fold (±0.49; *p* < 0.001) upregulated in those cultivated at LH for 48 h. No difference in *galectin-3* expression was verified in porcine corneas incubated at LH for 12 h (1.74 ± 0.41; *p* = 0.12) in comparison to the 12 h HH controls (Figure 3B). Upregulation of *occludin* and *galectin-3* expression was clearly induced in the porcine model incubated at LH for 24 h and 48 h.

### 2.4. Low Humidity Causes Porcine Corneal Cell Death

**Figure 4:**
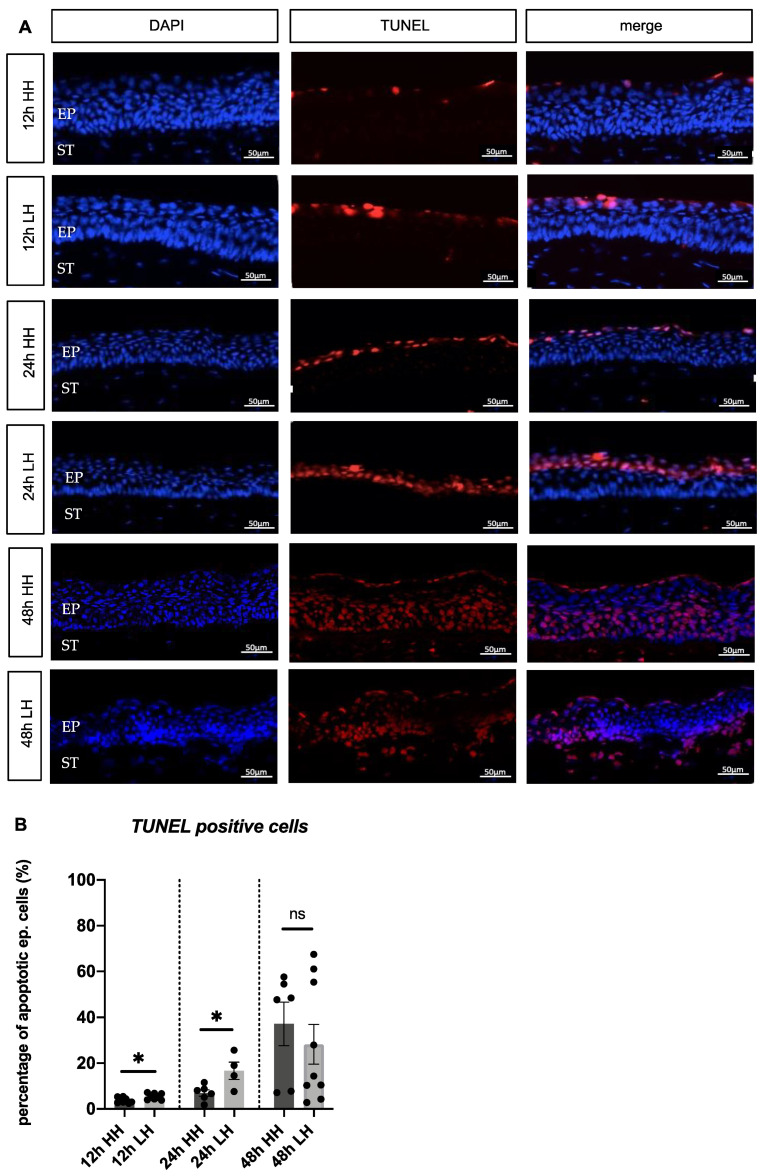
Incubation for 12 h and for 24 h at LH induced apoptosis in the corneal epithelium. (**A**) Representative images of porcine corneas incubated at LH for 12 h, 24 h and for 48 h and stained with TUNEL (red) and DAPI (blue) and controls incubated at HH for the same time-period. (**B**) Increased percentage of apoptotic cells in porcine corneas incubated at LH for 12 h and for 24 h in comparison to the HH samples (12 h LH = 5.38% ± 0.48/12 h HH = 3.87% ± 0.44 apoptotic cells; *p* = 0.036) (24 h LH = 16.69% ± 3.79/24 h HH = 6.98% ± 1.37 apoptotic cells; *p* = 0.0229) (*n* = 8 for 12 h LH/*n* = 8 for 12 h HH/*n* = 4 for 24 h LH/*n* = 6 for 24 h HH). No significant difference was observed between samples submitted to LH and HH for 48 h (48 h LH = 28.23% ± 8.67/48 h HH = 37.19% ± 9.53 apoptotic cells; *p* = 0.86) (*n* = 9 for 48 h LH/*n* = 6 for 48 h HH). LH: low humidity. HH: high humidity. EP: epithelium. ST: stroma. DAPI: 4′,6-diamidino-2-phenylindole. TUNEL: TdT-mediated dUTP-biotin nick end labelling. ns: not significant. Scale bar = 50 µm. The bars represent the mean values, and the SEM is shown. All data are shown as mean ±SEM. * *p* < 0.05.

To investigate whether the loss of epithelial cells and the reduced thickness in the model is caused by apoptosis, TUNEL staining was performed. After incubation for 12 h and for 24 h at LH, a higher percentage of apoptotic epithelial cells in comparison to the HH groups was obvious (Figure 4A). Quantitative analysis of apoptotic cells to overall cell number confirmed these findings (12 h LH= 5.38% ± 0.48/12 h HH= 3.87% ± 0.44 apoptotic cells; *p* = 0.036) (24 h LH = 16.69% ± 3.79/24 h HH = 6.98% ± 1.37 apoptotic cells; *p* = 0.0229) (Figure 4 B). Regarding the corneas incubated for 48 h, no significant difference was found in the LH group compared to the HH group (48 h LH = 28.23% ± 8.67/48 h HH = 37.19% ± 9.53 apoptotic cells; *p* = 0.86) (Figure 4A,B). As a result, the incubation of porcine corneas at LH for 12 h and for 24 h stimulated cell death.

### 2.5. Treatment with Dexamethasone Counteracts LH Effects but Only Partially with Hyaluronic Acid Eye Drops 

Incubation at LH for 24 h caused significant and robust effects of inflammation, corneal thinning, upregulation of tight junctions and glycocalyx markers and cellular apoptosis compared to 12 h and to 48 h incubation. Therefore, it was further investigated whether the inflammation and changes caused by LH could be reversed with eye drops currently used to treat DED. The eye drops used contained either dexamethasone 1.3 mg/mL (DexaEDO^®^, Bausch-Lomb, Heidelberg, Germany) (dexa) every 12 h or hyaluronic acid 0.2% (Artelac^®^ Splash EDO^®^, Bausch-Lomb, Heidelberg, Germany) (HA) every 6 h. 

Dexa is a corticosteroid that presents a therapeutic effect against inflammation and HA is an eye lubricant that helps moisten the cornea, consequently reducing inflammatory processes caused by DED. To verify whether inflammatory processes could be reversed, porcine corneas were divided in four groups: two incubated at LH, one with eye drop treatment and another without it, and two incubated at HH, one with eye drop treatment and another without it.

**Figure 5:**
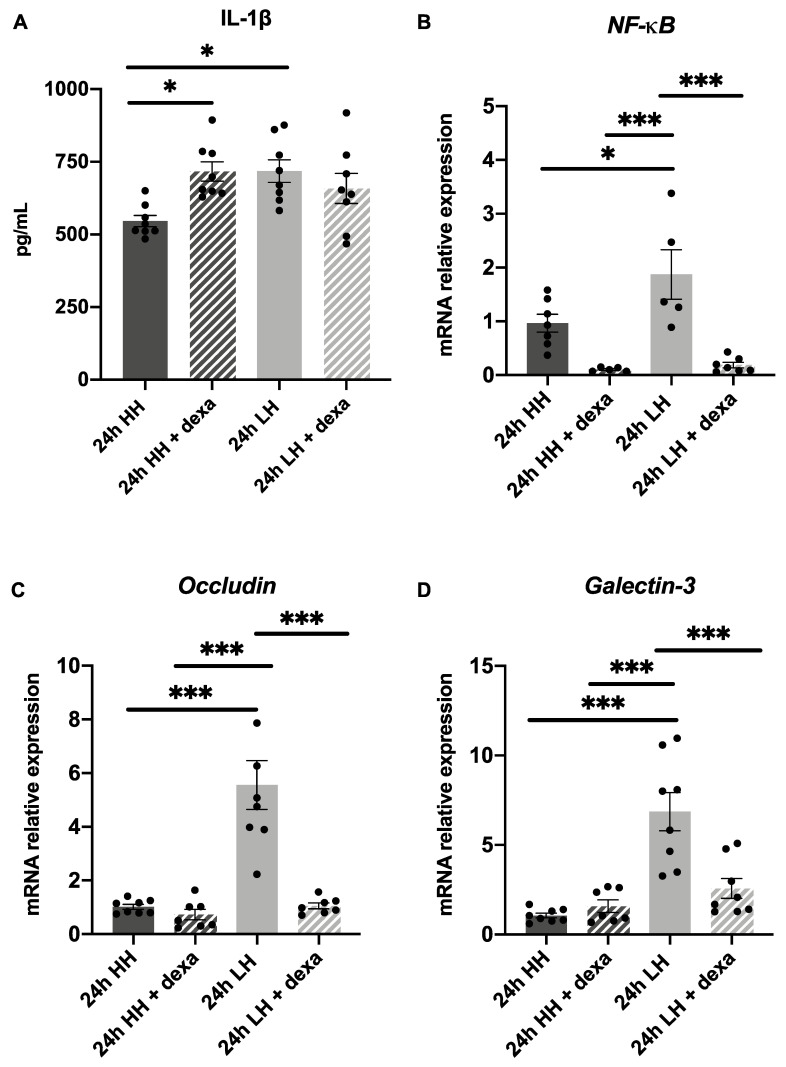
Dexamethasone (dexa) counteracts the effects caused by LH. (**A**) No significant difference in IL-1β secretion was observed in samples incubated at LH + dexa in comparison to LH group (LH + dexa= 658.10 pg/mL ± 51.65/LH= 718.10 pg/mL ± 38.85; *p* = 0.68) (*n* = 8 for LH/*n* = 8 for LH + dexa/*n* = 8 for HH/*n* = 8 for HH + dexa). (**B**) A significant inhibition of increase in *NF-κB* mRNA expression was observed at the LH + dexa group (0.18 ± 0.05) in comparison to LH group (1.87 ± 0.46 *p* < 0.001). (**C**) The increase in *occludin* mRNA expression due to LH was significantly prevented by dexa (1.05 ± 0.11), in comparison to LH group (5.56 ± 0.91; *p* < 0.001). (**D**) LH significantly induced mRNA expression of *galectin-3* (6.85 ± 1.07; *p* < 0.001). This effect could be prevented by the treatment with dexa (2.57 ± 0.55; *p* < 0.001). *n* = 5–8 for LH/*n* = 7–8 for LH + dexa/*n* = 7–8 for HH/*n* = 5–7 for HH + dexa. LH: low humidity. HH: high humidity. Dexa: dexamethasone. The bars represent the mean values, and the SEM is shown. * *p* ˂ 0.05 *** *p* ˂ 0.001.

To evaluate the effect of dexa treatment on the LH induced inflammation, the secretion of IL-1β and the mRNA expression of *NF-κB, occludin* and *galectin-3* was investigated. Comparable to the prior results (Figure 2A), an augmentation of IL-1β was found in samples submitted to LH (718.10 pg/mL ± 38.85) in comparison to the ones incubated at HH (546.00 pg/mL ± 19.49; *p* = 0.02) (Figure 5A). However, no significant difference in IL-1β secretion was observed in samples incubated at LH and treated with dexa, in comparison to the ones incubated at LH (LH + dexa = 658.10 pg/mL ± 51.65/ LH = 718.10 pg/mL ± 38.85; *p* = 0.68) (Figure 5A). Treatment with dexa in corneas incubated at LH could significantly prevent an increase in *NF-κB* mRNA (0.18 ± 0.05; *p* < 0.001) in comparison to the ones incubated at LH (1.87 ± 0.46) (Figure 5B). Similar to previous results (Figure 3A), an upregulation of *occludin* mRNA (5.56 ± 0.91; *p* < 0.001) was observed in samples incubated at LH in comparison to the ones submitted to HH (Figure 5C). Furthermore, a significant inhibition of *occludin* mRNA expression (1.05 ± 0.11; *p* < 0.001) was observed in corneas incubated at LH + dexa in comparison to the ones incubated at LH (Figure 5C). In addition, the upregulation of *galectin-3* mRNA expression (LH: 6.85 ± 1.07; *p* < 0.001) in samples incubated at LH could be significantly counteracted by treatment with dexa (2.57 ± 0.55; *p* < 0.001) (Figure 5D). These findings confirm that dexa counteracts the LH-triggered mRNA increase in *NF-κB, occludin* and *galectin-3* and can also reduce inflammatory processes at the transcriptional level in the ex vivo model.

**Figure 6:**
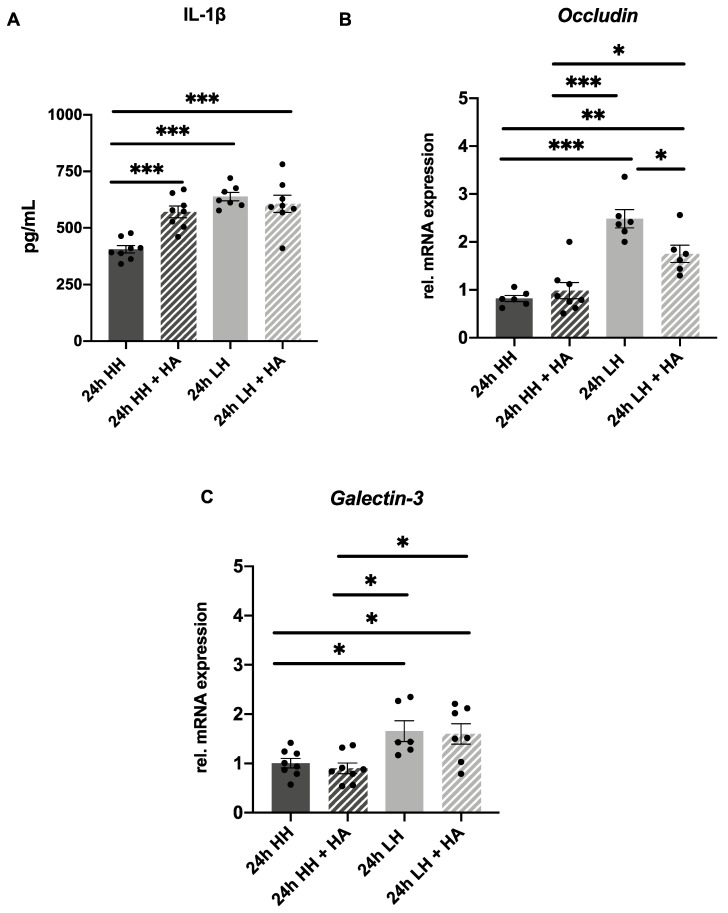
Hyaluronic acid (HA) partially counteracts the effects caused by LH. (**A**) No significant difference at IL-1β secretion was shown between LH + HA group in comparison do LH group (LH + HA = 607.00 pg/mL ± 37.54/LH = 638.60 pg/mL ± 18.68; *p* = 0.84). An increase in IL-1β was found in samples exposed to LH (638.60 pg/mL ± 18.68), to LH + HA (607.00 pg/mL ± 37.54) and to HH + HA (571.20 pg/mL ± 25.41) in comparison to the ones incubated at HH (406.10 pg/mL ± 16.45; *p* < 0.001) (*n* = 7 for LH/*n* = 8 for LH + HA/*n* = 8 for HH/*n* = 8 for HH + HA). (**B**) A significant hindering of increase in *occludin* mRNA expression was observed at the LH + HA group (1.75 ± 0.18) in comparison to LH (2.48 ± 0.19; *p* = 0.03). (**C**) No significant difference in *galectin-3* expression was shown at the LH + HA group (1.60 ± 0.21) in comparison to LH group (1.66 ± 0.21; *p* = 0.04). *n* = 6 for LH/*n* = 6–7 for LH + HA/*n* = 6–8 for HH/*n* = 8 for HH + HA. LH: low humidity. HH: high humidity. HA: hyaluronic acid. The bars represent the mean values, and the SEM is shown. * *p* ˂ 0.05 ** *p* ˂ 0.01 *** *p* ˂ 0.001.

Next, some markers were tested to investigate whether treatment with HA could also counteract the effects caused by LH. An increase in IL-1β was found in samples exposed to LH (638.60 pg/mL ± 18.68), to LH + HA (607.00 pg/mL ± 37.54) and to HH + HA (571.20 pg/mL ± 25.41) in comparison to the ones incubated at HH alone (406.10 pg/mL ± 16.45; *p* < 0.001) (Figure 6A). Again, in this sample series, an upregulation of *occludin* mRNA expression 2.48 ± 0.19; *p* < 0.001) was observed in corneas incubated at LH in comparison to the ones submitted to HH only (Figure 6B). Furthermore, treatment with HA could significantly reduce the increase in *occludin* mRNA expression in samples submitted to LH and treated with HA (1.75 ± 0.18; *p* = 0.03) compared to the ones incubated at LH without treatment (2.48 ± 0.19) (Figure 6B). In addition, an upregulation of *galectin-3* mRNA expression was observed in samples incubated at LH (1.66 ± 0.21; *p* = 0.04) in comparison to the ones submitted to HH (Figure 6C), but no counteracting effect of HA treatment could be detected (1.60 ± 0.21 *p* > 0.99). This finding suggests that HA was able to partially counteract the effects of incubation at LH at a transcriptional level.

## 3. Discussion

### 3.1. An Ex Vivo Dry Eye Model Is Proposed

DED has a high prevalence and is mainly treated with artificial tears [3,34]. There is a high need for developing new therapies for DED, as the worldwide disease burden is high. However, for testing new therapies, robust models are needed. 

Currently, in vivo models for DED have already been developed, but they are expensive and the use of animals for research purposes should be avoided due to ethical issues [14,15]. In addition, previous ex vivo models were developed with the need to use, for example, a pumping system to simulate tears or a mechanical arm to simulate blinking [16,35]. However, such models are difficult to reproduce and require special tools.

The current work was carried out to establish a cheap and robust ex vivo dry eye model which will not only make it possible to test new therapies efficiently, but also reduce the use of animals in research. Moreover, porcine eyes have a similar morphology to the human eye [36] and similar size [37]. It has been described that the structure of porcine corneas and human corneas are similar regarding to the morphology, both species present corneal epithelium constituted of stratified, non-keratinized squamous cell layer, Bowman’s membrane, stroma with collagen fibers, Descemet membrane and corneal endothelia [38] so a simple transfer of knowledge is possible, however, the presence of Bowman’s membrane in porcine corneas is not a consensus between authors [36,38].

LH is a well-known risk factor for DED [39]. Humidity of 30% has already been used previously to induce dry eye disease in an in vivo model and in an in vitro model [40]. To verify the effect of 30% humidity, corneal epithelial tissues were incubated at 30% humidity for different durations of time. Incubation for 24 h in LH (37 °C and 30% humidity) induced changes similar to DED, i.e., the characteristic epithelial features normally found in DED. This is in accordance with previous studies, that also related such conditions to be the ideal ones [40].

It is important to point out that the air-lifting technique that was applied to culture the porcine corneas, allowing a thin layer of medium to humidify the corneal surface and the air contact above it, presents the advantage of maintaining the physiological barrier function of the corneal epithelium. This technique was previously described to be superior for the cultivation of in vitro corneal epithelial cells when compared to the non-air-lifting technique, in which the cells are completely submerged in culture medium [41].

To better assess the histological changes in the model, the epithelial thickness was measured, and disruption of the epithelial cells was analyzed with HE staining. It has been proposed in previous studies that LH induces a reduction of epithelial thickness, and it is well known that disruption of the epithelial cells is an important sign of DED [42,43]. In this study, porcine corneas exposed to stress at LH for 12 h exhibited significant reduction in epithelial thickness, but epithelial damage was mild. However, corneas incubated at LH for 24 h showed epithelial damage, with detachment of the superficial cells of the cornea (Figure 1A) and significant reduction of the epithelial thickness (Figure 1B). Similarly, Pelegrino et al. reported that LH stress changes the corneal barrier function and leads to a reduction of epithelial thickness [40,43]. 

Porcine corneas exposed to stress at LH for 48 h showed severe epithelial damage, with detachment of not only the superficial cells of the cornea, but also internal layers (Figure 1A). However, no statistically significant reduction of the epithelial thickness between groups was found (Figure 1B). These facts indicate that incubation of porcine corneas for a period longer than 24 h may cause epithelial damage not only to stressed samples, but also to controls. Due to the origin, collection, and transport of the porcine eyes, which are not comparable to the procedure performed on human donor eyes, there are pre-damages that make a longer cultivation time difficult. Irreversible cell lesions were also observed in the control groups after 48 h, which only intensified after 72 h (data not shown). However, nonetheless, after 24 h, damage of the superficial corneal epithelial cells and other DED markers increased, and the effect could be counteracted. Moreover, in human corneas from donors that are incubated at cornea banks, damage of the corneal epithelia also occurs in death-to-preservation time intervals longer than 6 h [44].

IL-1β, a cytokine that is an important mediator of the inflammatory response, is more highly secreted in the tear film of patients with DED [45,46]. Na et al. also described a correlation between the augmentation in concentration of IL-1β in tears of patients with DED and the severity of the disease [47]. Similarly, Okanobo et al. reported that DED-associated inflammation leads to an increase in IL-1β, but that could be suppressed in their in vivo model with the use of topical Interleukin-1 receptor antagonist (IL1-Ra) [48]. To verify whether the current model also exhibits the same increased secretion, IL-1β levels were measured. An elevation of the inflammatory cytokine IL-1β was found in the stressed corneas when incubated at LH for 24 h and for 48 h (Figure 2A). This confirms that this important DED-associated inflammation marker is also increased in samples incubated under the desiccation conditions in the proposed ex vivo model.

NF-κB is well known as a transcription factor that is involved in the cellular response to stimuli such as stress, cytokines, and free radicals. Moreover, it stimulates the production of TNF-α and IL-1β, which ultimately activate MMP9. Because of this, mRNA expression of these pro-inflammatory markers was investigated in this study. *NF-κB* was upregulated in porcine corneas incubated at LH for 12 h and 24 h (Figure 2C). Furthermore, mRNA of *IL-1β* and *TNF-*α was upregulated in corneas stressed for 48 h (Figure 2B,D). According to the literature, elevated pro-inflammatory cytokines such as IL-1β and TNF-α occur in DED [49,50].

Moreover, environmental stress, such as LH and high osmolarity, leads to activation of NLRP3. Activated NLRP3 ultimately promotes maturation and secretion of IL-1β [13]. Because of this, mRNA expression of *NLRP3* was also checked in this study, and an upregulation in corneas submitted to LH for 48 h was observed (Figure 2E).

IFN-γ has been described as a biomarker for DED [24,51]. In accordance with this, IFN-γ was increased in the media of porcine corneas incubated at LH for 48 h. Furthermore, the pro-inflammatory cytokine MIF and the pro-inflammatory and pro-angiogenic cytokine ang-1 were also increased in the media of porcine corneas cultivated at LH for 48 h. An augmentation of a pro-angiogenic cytokine, FGF21, was observed in the media of samples stressed at LH for 24 h. These data support our hypothesis that inflammatory mechanisms are stimulated in our porcine model. The fact that erythropoietin, galectin-9 and IFN-β, which are anti-inflammatory cytokines, were increased in the media of samples incubated at LH for 24 h might indicate a protection mechanism against LH effects (Table 1).

The matrix metalloproteinase MMP9 is responsible for the cleavage of the epithelial basement membrane and tight junctions’ proteins, such as occludin [40,52]. Active MMP9 also cleaves galectin-3, a component of the epithelial glycocalyx [7]. A significant upregulation of *occludin* and *galectin-3* mRNA expression in samples cultivated at LH for 24 h and 48 h was observed (Figure 3A,B). These facts corroborate the hypothesis that upregulation of mRNA expression of *occludin* and *galectin-3* can be interpreted as an early marker that acts as a positive signal to stimulate their production. Meloni et al. also reported the upregulation of *occludin* mRNA expression as an early biomarker for irritation. It was observed that strong irritants cause this upregulation, which can be correlated with early signs of cell defense and recovery potential [40,43].

To confirm that the loss of epithelial cells in the model is due to cell death, TUNEL assay was performed. Porcine corneas cultured at LH for 12 h and 24 h presented a higher percentage of apoptotic cells in comparison to controls (Figure 4A), which can be confirmed by the quantification of percentage of apoptotic epithelial cells (Figure 4B). The fact that no significant difference was observed in samples incubated at LH for 48 h corroborates the hypothesis that 48 h is too long period of incubation for the porcine corneas.

**Figure 7:**
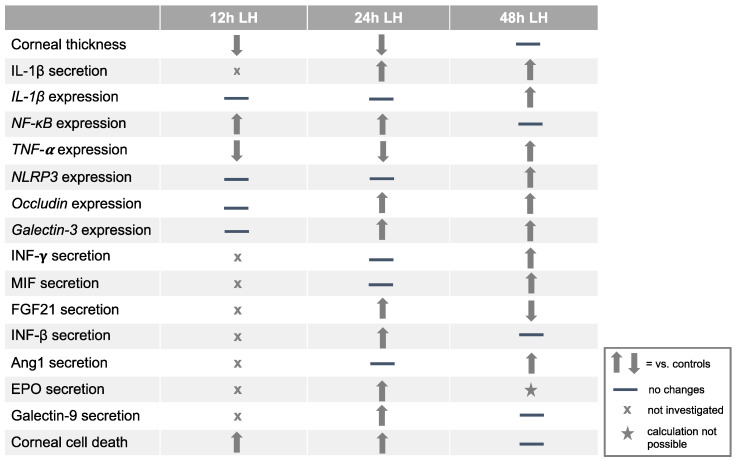
Table summarizing the results of ex vivo porcine corneal incubation at LH for 12 h, 24 h and 48 h. An increase or decrease in each factor was considered when the comparison between LH and HH was statistically significant, or in the case of the Cytokine Antibody Array when the ratio LH/HH was lower than 0.5 or higher than 1.5.

In summary, incubation at LH for 24 h is proposed as the best time point to incubate the dry eye model. At this time point, damage of the superficial corneal epithelial cells and thinning of the corneal epithelium were demonstrated. Moreover, increased secretion of IL-1β and upregulation of *NF-κB*, *occludin* and *galectin-3* were confirmed. Cell death was also induced in corneas cultivated at LH for 24 h, as expected (Figure 7).

### 3.2. Treatment with Dexamethasone Can Reverse Effects of Low Humidity and with Hyaluronic Acid Might Partially Reverse

The use of artificial tears composed of hyaluronic acid (HA) to moisten the cornea and the use of corticosteroids, such as dexa, to reduce inflammation is widespread. Artificial tears are routinely used for the treatment of DED. However, it is now clear that inflammation plays an important role in the pathophysiology of this disease. Because of that, corticosteroids, such as dexa, are used for the treatment of DED, inhibiting inflammatory mediators [18]. A hindering of the increase in *NF-κB* mRNA expression in porcine corneas incubated at LH and dexa treatment in comparison to those only incubated at LH was demonstrated (Figure 5B). According to the literature, glucocorticoids act in different pathways to inhibit NF-κB [18,53].

It has already been described that dexa hinders corneal epithelial injury [17]. Here, it was demonstrated that treatment with dexa could inhibit LH-induced mRNA expression of *occludin* and *galectin-3* (Figure 5C,D). These facts support the hypothesis that dexa can reverse the inflammatory and corneal damage caused by LH.

Artificial tears are usually the first therapeutic choice for treating DED, because they help to improve the symptoms and to make the tear film more stable [15]. It was verified that porcine corneas incubated at LH and treated with HA presented a prevention of increase in the mRNA expression of *occludin* in comparison to the ones submitted to LH (Figure 6B), which indicates that the damage caused by LH might be reversed with the treatment with HA. The fact that no significant difference in *galectin-3* mRNA expression was found in the LH + HA group in comparison to the LH group (Figure 6C) indicated that the effects of the treatment with HA are less prominent than those of the treatment with dexa, or that it might take a longer period to achieve a better therapeutic effect. In accordance with our findings, Meloni et al. described that in their 24 h in vitro dry eye model, only a corticosteroid was able to counteract the effect of inflammatory genes, whereas artificial tears were not able to do so [40]. Furthermore, we observed that porcine corneas treated with HA at LH or HH presented an increased secretion of IL-1β in comparison to the HH group (Figure 6A). It has been described that low-molecular-weight HA can trigger inflammation, increasing pro-inflammatory cytokine gene expression of *IL-1*β [54]. Aragona et al. analyzed 18 commercially available artificial tears and determined their molecular weight. Artelac^®^ Splash presents an average molecular weight of 533 kDa, so Aragona et al. classified it as medium-molecular weight HA. However, eye drops with molecular weight lower than 500 kDa were classified as low-molecular weight [55]. Taking these facts into consideration, we hypothesize that the increased IL-1β secretion in our model might have been caused by the eye drops containing HA used in our research.

Moreover, we speculate that the increased secretion of IL-1β in the samples incubated at LH and treated with dexa or HA was not prevented by these treatments in the porcine model because 24 h was too short a period to induce such molecular modification. However, changes at the transcriptional level were clearly demonstrated, especially when the porcine model was treated with dexa.

## 4. Materials and Methods

### 4.1. Preparation of Porcine Corneas

**Figure 8:**
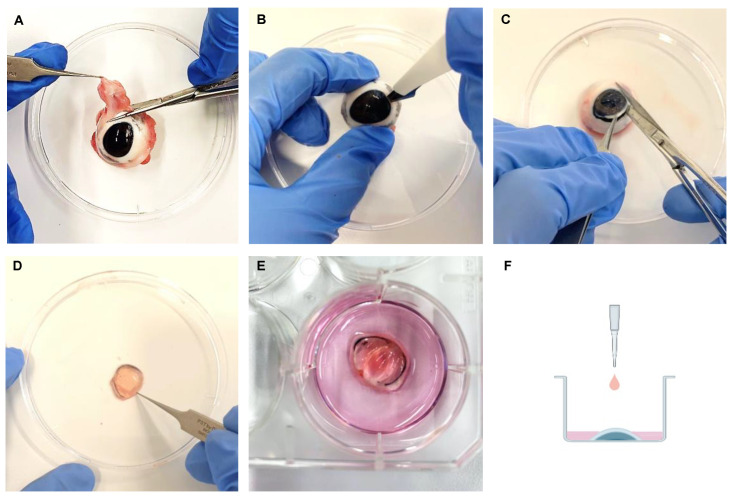
Porcine corneal extraction. (**A**) Muscles and tissues around the eye were removed. (**B**–**D**) To extract the cornea a cut was made around it with a scalpel and finalized with scissors. (**E**) A thin part of the sclera was left around the cornea. (**F**) The corneas were incubated in a 6-well plate, facing upwards, and the appropriate media was added. (**F**) Schematic figure depicts air-lifting technique, porcine cornea in a 6 well-plate facing upwards and culture media in pink meets the corneal epithelium surface.

Porcine eyes were obtained from a local abattoir, in Balingen, Germany. After euthanization of the pigs, the eyes were enucleated and cooled at 4 °C until being used. Before the experiment, the pig eyes were brought to room temperature and muscles and tissues around the eye were discarded. The eyes were disinfected in iodine 5% (*v/v*) diluted in distilled water for 5 min. After that, the eyes were washed 5 times in Dulbecco’s Phosphate Buffered Saline (PBS) (Sigma-Aldrich, Taulfkirchen, Germany) with 2% penicillin-streptomycin (Thermo-Fischer, Karlsruhe, Germany). The pig corneas were then extracted from the eyes with scalpel, scissors and forceps, a cut was made around the cornea, leaving a thin part of the sclera around it. The cornea samples were placed on 6-well plates (clear Falcon^®^ Corning, Glendale, AZ, USA) with the cornea facing upwards (Figure 8).

### 4.2. Ex Vivo Dry Eye Model: Incubation at Low Humidity for 12 h, 24 h or 48 h

The corneas were divided in two groups and each cornea received 800 µL of media (Culture Media II (Biochrom GmbH, Berlin, Germany)) supplemented with 2% penicillin-streptomycin (Thermo Fischer, Karlsruhe, Germany). This amount of media met the surface of the porcine corneal epithelium, so that the media moisturized the apical epithelium cells and the air made contact with this thin layer of media [41] (Figure 8F). Corneas were cultivated in a 6-well plate (clear Falcon^®^ Corning, Glendale, AZ, USA) at 37 °C and 30% humidity, while the controls were cultivated at 37 °C and 95% humidity for 12 h or for 24 h. To investigate a longer period of incubation at low humidity (LH), corneas were cultivated at 37 °C and 30% humidity for 48 h, while the control group, was cultivated at 37 °C and 95% humidity for the same time. 

### 4.3. Production of Cryosections

After each incubation time, the corneas were frozen with Tissue-Tek (Sakura Finetek, Torrance, CA, USA) in liquid nitrogen and stored at −28 °C. Cryosections were prepared with a thickness of 12 μm, and three sections each of a cornea were applied to a microscope slide (R. Langenbrinck GmbH, Emmendingen, Germany) and stored unfixed at −28 °C until further processing.

### 4.4. Periodic Acid–Schiff Staining

Periodic acid–Schiff (PAS) staining was used to verify the glycocalyx content of the tissue samples. The cryosections were fixed with ice-cold methanol (Honeywell, Offenbach, Germany) for 10 min and the slides were incubated in 1% periodic acid (Carl Roth, Karlsruhe, Germany) for the next 10 min. The acid was washed with running water and the samples were incubated for 10 min with Schiff’s reagent (Carl Roth, Karlsruhe, Germany) and washed under running water again. The slides were placed in a container filled with Harris Hematoxylin Solutions (HHS) (Sigma-Aldrich, Taufkirchen, Germany) for 10 min and differentiated with hydrochloric acid–alcohol solution (AppliChem, Darmstadt, Germany) for 3 s. After that, they were washed and left in the water for 10 min to remove the excess staining. The samples were dehydrated first with 2 times 96% (*v/v*) ethanol (VWR, Darmstadt, Germany) and then with 2 times 99% (*v/v*)). The sections were “dipped” into a row of xylene (VWR, Darmstadt, Germany) (four vessels filled with xylene) and embedded with Eukitt (Sigma-Aldrich, Taufkirchen, Germany).

### 4.5. Hematoxylin-Eosin (HE) Staining 

The cryosections were fixed in ice-cold methanol (Honeywell, Offenbach, Germany) for 10 min and washed in distilled water twice for 1 min. Afterwards, they were stained with HHS (Sigma-Aldrich, Taufkirchen, Germany) for 10 min and washed twice with water. Next, the slides were differentiated in hydrochloric acid-alcohol solution (AppliChem, Darmstadt, Germany) for 3 s, washed with water for 10 min and placed in Eosin Y (Sigma-Aldrich, Taufkirchen, Germany) for 2 min. After that, the cryosections were dehydrated using an ascending ethanol (VWR, Darmstadt, Germany) series (70% (*v/v*), 2 times 96% (*v/v*), 2 × 99% (*v/v*)) and in a series of 4 times xylene (VWR, Darmstadt, Germany). Finally, the samples were embedded with Eukitt (Sigma-Aldrich, Taufkirchen, Germany).

### 4.6. Histological Analysis 

The thickness of the porcine corneal epithelium was measured with an image analyzing system (ZEN Imaging Software, Carl Zeiss, Oberkochen, Germany). To reduce test variability, the mean of six measures per picture and six pictures of 200-fold magnification per sample was used for analysis of porcine corneas. The Zeiss Axio Imager Z1 Apotome Microscope with Mrm digital camera (Carl Zeiss, Oberkochen, Germany) was used for imaging.

### 4.7. Quantitative Real-Time PCR

The expression of inflammation markers, like *NF-κB, TNF-α, IL-1β* and *NLRP3*, was analyzed. The mRNA expression of tight junction’s marker *occludin* and the glycocalyx marker *galectin-3* was also investigated in the porcine model (Table 2). Briefly, after incubation, the corneas were cut into small pieces and incubated in 800 µL Lysis Buffer (Miltenyi Biotec, Köln, Germany) for 1 h at 37 °C on a shaker. The mRNA was isolated from porcine cornea explants and reverse transcribed using the MultiMACS mRNA and cDNA synthesis was done with cDNA Synthesis Kit on the MultiMACS™ M96 Separator (Miltenyi Biotec, Köln, Germany) according to the manufacturer’s protocol. After cDNA synthesis, a quantitative real-time PCR (qRT-PCR) was performed using the SsoAdvanced Universal SYBR^®^ Green Supermix (Bio-Rad, Feldkirchen, Germany) in a thermal cycler (Bio-Rad CFX96™ Real-Time System, Bio-Rad, Feldkirchen, Germany), as described previously [56]. *β-actin* (*ACTB*) and *Ribosomal protein L 4 (RPL4)* were used as housekeeping genes. 

### 4.8. IL-1β ELISA 

IL-1β levels in the cultivation medium of porcine cornea explants were quantified by using the Lumit^TM^ IL-1β Human Immunoassay. #W6010 (Promega, Walldorf, Germany) according to manufacturer’s protocol. Briefly, 50 µL of media was applied in duplicate in a 96-well plate (white/clear Falcon^®^ Corning, Glendale, AZ, USA) together with 50 µL antibody conjugate (Promega, Walldorf, Germany) diluted 1:500 in media. The plate was incubated at 37 °C for 1 h. Afterwards, a 20-fold dilution of Lumit^TM^ Detection Substrate B in Lumit^TM^ Detection Buffer B (Promega, Waldorf, Germany) was performed and 25 µL was added to the samples. The luminescence was measured with a Tecan Spark (Tecan, Männedorf, Switzerland).

### 4.9. TdT-Mediated dUTP-Biotin Nick End Labelling (TUNEL) Assay

Corneal epithelial cell death was analyzed using fluorescent in situ terminal deoxynucleotidyl transferase (TdT)-mediated uridine 5′-triphosphate-biotin nick end labeling (TUNEL assay, Roche Diagnostics, Mannheim, Germany). TUNEL assay was performed according to the manufacturer’s protocol, as described previously [57]. To stain the nuclei, 4′,6-diamidino-2-phenylindole (DAPI) staining was performed. The Zeiss Axio Imager Z1 Apotome Microscope with Mrm digital camera (Carl Zeiss, Oberkochen, Germany) was used for imaging. A cell count was performed with semi-automated counter software ImageJ (ImageJ, U. S. National Institutes of Health, Bethesda, MD, USA, https://imagej.nih.gov/ij/, 30 November 2021). To reduce test variability, the mean of six picture per sample of 200-fold magnification was used for analysis (*n* = 8 for 12 h LH/*n* = 8 for 12 h HH/*n* = 4 for 24 h LH/*n* = 6 for 24 h HH/*n* = 9 for 48 h LH/*n* = 6 for 48 h HH).

### 4.10. Porcine Cytokine Antibody Array

For the simultaneous detection of multiple cytokines in the different culture media (HH vs. LH), a semi-quantitative Porcine Cytokine Array was performed (RayBio^®^ C-Series Porcine Cytokine Array 1 AAP-CYT-1-4 (RayBiotech, Peachtree Corners, GA, USA)). The media from six samples per condition (24 h HH/ 24 h LH/ 48 h HH/ 48 h LH) were pooled to form a total of 500 µL per array and the assay was performed according to manufacturer’s protocol. Briefly, a total of 500 µL of culture media per condition was added to the antibody arrays and incubated at room temperature for 5 h. Washing steps were conducted, and 500 µL of Biotinylated Antibody Cocktail was pipetted into each well containing an antibody array and incubated overnight at 4 °C. After a second washing step, 2 mL HRP-Streptavidin was added into each well and incubated for 2 h at room temperature. A third washing step was carried out, and chemiluminescence detection was performed. A comparison of signal intensities for individual antigen-specific antibody spots among arrays images was used to determine relative differences in expression levels of each analyte. To perform data analysis, the background was subtracted from the raw numerical densitometry data and the data were normalized to the positive control signals. Then, a fold increase or decrease in the signal intensity of each cytokine secreted at LH media was calculated in relation to HH. It was considered a fold increase when higher than 1.5 and a fold decrease when lower than 0.5.

### 4.11. Ex Vivo Dry Eye Model: Treatment with Dexamethasone and Hyaluronic Acid

Porcine corneas were divided into four groups, and each cornea received 800 µL of media (Culture Media II (Biochrom GmbH, Berlin, Germany)) supplemented with 2% penicillin-streptomycin (Thermo Fischer, Karlsruhe, Germany). To investigate whether the damage caused by LH could be reversed, samples were treated with one drop of dexamethasone 1.3 mg/mL (DexaEDO^®^, Bausch-Lomb, Heidelberg, Germany) every 12 h or with one drop of hyaluronic acid 0.2% (Artelac^®^ Splash EDO^®^, Bausch-Lomb, Heidelberg, Germany) every 6 h. Corneas were cultivated at 37 °C and 30% humidity, with or without treatment, while the controls were cultivated at 37 °C and 95% humidity, with or without treatment, for 24 h. 

### 4.12. Statistical Analysis

Statistical analysis was performed with GraphPad Prism^TM^ Version 8.2.1 (GraphPad Software, San Diego, CA, USA). The Student’s *t*-test was used to compare two groups, when normality was confirmed. The Student’s *t*-test with Welch’s correction was used when normality was certified, but variance was unequal. For multiple comparisons, one-way ANOVA was used, followed by a Tukey’s post hoc test for unequal or equal groups, when normality was attested. Kruskal Wallis with Dunn’s post hoc test was applied for multiple comparisons when the distribution was not normal. Results are presented as mean ± standard error of the mean (SEM). A *p*-value < 0.05 was considered to be statistically significant. The level of significance was set to * *p* < 0.05, ** *p* < 0.01 and *** *p* < 0.001.

## 5. Conclusions

Here, a novel, cheap and easy-to-reproduce ex vivo corneal model for DED is introduced. Incubation of porcine corneas at 30% humidity and 37 °C stimulated similar changes like the ones found in DED. Furthermore, 24 h incubation was the superior time point for this induction in comparison to 12 h and 48 h incubation. HA was able to partially counteract the effects of incubation at LH. Dexa was proven to reduce the inflammatory effects caused by LH in this dry eye ex vivo model.

## Figures and Tables

**Table 1:** Cytokines secreted in media of porcine corneas incubated at LH for 24 h and 48 h. Signal intensity ratio of each cytokine secreted at LH media was calculated in relation to HH and fold increase or decrease is demonstrated. LH: low humidity. HH: high humidity. -: ratio could not be calculated when signal intensity was lower than background.

Cytokine	Ratio 24 h LH/HH	Ratio 48 h LH/HH	Function
Erythropoietin	11.59	-	Decreases apoptosis and inflammation [20]
Galectin-9	3.29	0.80	Anti-inflammatory/anti-angiogenic [21]
Interferon-beta	4.92	1.25	Anti and pro-inflammatory [22]
Granulocyte-macrophage colony-stimulating factor	1.44	-	Pro-inflammatory [23]
Interferon-gamma	1.36	2.67	Pro-inflammatory [24]
Macrophage migration inhibitory factor	1.01	1.68	Pro-inflammatory [25]
Chemokine (C-C motif) ligand 3-like 1	0.71	1.41	Pro-inflammatory [25]
Interleukin 22	0.01	0.63	Anti and pro- inflammatory [26]
Osteoprotegerin	1.43	-	Angiogenesis [27]
Fibroblast growth factor 21	1.54	0.44	Angiogenesis [28]
Angiopoietin-1	0.71	2.10	Angiogenesis/Inflammation [29]
Vascular endothelial growth factor	0.44	1.41	Angiogenesis/Inflammation [30]
Tissue inhibitor of metalloproteinases 2	0.83	3.37	Inhibits apoptosis and induces corneal epithelial cell proliferation [31]
Transforming growth factor alpha	0.12	0.56	Cell proliferation, differentiation, and development [32]

**Table 2:** Primer pairs in 5′-3′directions used for a quantitative real-time PCR.

Primer	Forward	Reverse
*NLRP3*	CAGCACGAACCAGAATCTCA	AGCAGCAGTGTGATGTGAGG
*ACTB*	CACGCCATCCTGCGTCTGGA	AGCACCGTGTTGGCGTAGAG
*NF-κB*	AGGATGGGATCTGCACTGTC	ATCAGGGTGCACCAAAAGTC
*Occludin*	TCGGACTATGCGGAGAGAGT	TTTGAAGACGCCTCCAAGTT
*Galectin-3*	CTGGAAAACCAAACCCTCAA	CCAGGATAACCAGGTGCTGT
*TNF-α*	CCCCTGTCCATCCCTTTATT	AAGCCCCAGTTCCAATTCTT
*IL-1β*	CAGCCATGGCCATAGTACCT	CAGCCATGGCCATAGTACCT
*RPL4*	CAAGAGTAACTACAACCTTC	GAACTCTACGATGAATCTTC

## Data Availability

All data are provided within the main text. Further data inquiries are available from the authors upon reasonable request.
